# Angina Bullosa Hemorrhagica: A Rare and Interesting Presentation

**DOI:** 10.7759/cureus.23335

**Published:** 2022-03-20

**Authors:** Okelue E Okobi, Endurance O Evbayekha, Ujunwa Ebili, Uchechukwu O Ogbonna, Faithful Ogundiran, Imoh L Ebong

**Affiliations:** 1 Family Medicine, Lakeside Medical Center, Belle Glade, USA; 2 Internal Medicine, Stella Obasanjo Isolation Center, Benin, NGA; 3 Pediatrics, Premier Specialists Medical Center, Lagos, NGA; 4 Surgery, Barau Dikko Teaching Hospital, Kaduna, NGA; 5 Surgery, All Saints University School of Medicine, Roseau, DMA; 6 Internal Medicine, University of Ghana School of Medicine and Dentistry, Accra, GHA

**Keywords:** blood-filled blisters, mucosal blisters, oral mucosa, hemorrhagica, angina bullosa

## Abstract

Angina bullosa haemorrhagica (ABH) is a benign disorder of the oral mucosa. Patients present with blood-filled blisters in the oral cavity that are not associated with bleeding disorders. These blisters can sometimes be painful. This case report aims to highlight the features of ABH, which will help to distinguish it from other oral diseases and improve the quality of care for patients with this rare disease.

## Introduction

Angina bullosa haemorrhagica (ABH) is a term used to describe benign sub-epithelial oral mucosal blisters filled with blood not attributable to a systemic disorder or hemostatic dysfunction. ABH is a disorder of the oral cavity [1].

Clinically, blood-filled blisters are seen on the oral mucosa [2]. These blisters present a color ranging from dark red to purple and may cause some discomfort [3]. ABH mainly affects the soft palate of elderly adults, although occasionally these lesions may occur in the anterior pillar of the fauces, epiglottis, arytenoids, pharyngeal wall, and esophagus [3,4]. While the origin of ABH is unknown, the most widely accepted hypothesis is mild trauma, mainly during food ingestion [3,5]. Generally, it is an infrequent condition with a favorable resolution in a few days, and an overall good prognosis [1,4,5]. ABH is still poorly documented in the literature, and its etiology remains uncertain [4].

## Case presentation

A 76-year-old black unemployed male with a history of hypertension and benign prostatic hypertrophy presented to the hospital with spontaneous oral lesions. He noticed a palpable mass in the roof of his mouth approximately five minutes after he had his lunch. He described it as mildly painful and soft. He denies trauma or the use of artificial dentures. He says the lesion rapidly grew more prominent and felt like “it was moving towards the back of the throat and choking him,” prompting him to present to the hospital. He denies a history of similar symptoms, and he also denies a history of bleeding disorder. A coagulation panel done had all parameters within normal limits. He takes tamsulosin for his prostate enlargement and candesartan for his hypertension. He does not drink or smoke. The review of systems was not significant. 

On examination, vitals revealed blood pressure of 147/90 mmHg, heart rate of 98 beats per minute, respiratory rate of 16 cycles per minute, and oxygen saturation of 98% on room air.

The patient received a dose of methylprednisolone 125 mg IV stat and was admitted overnight for observation and airway monitoring. The lesion improved the following morning. The patient was discharged for outpatient follow-up.

**Figure 1:**
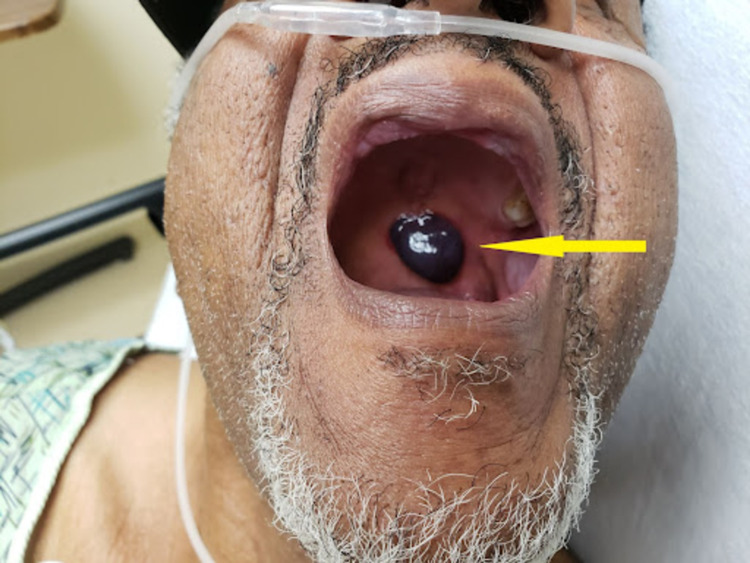
Arrows pointing to the lesion at presentation

**Figure 2:**
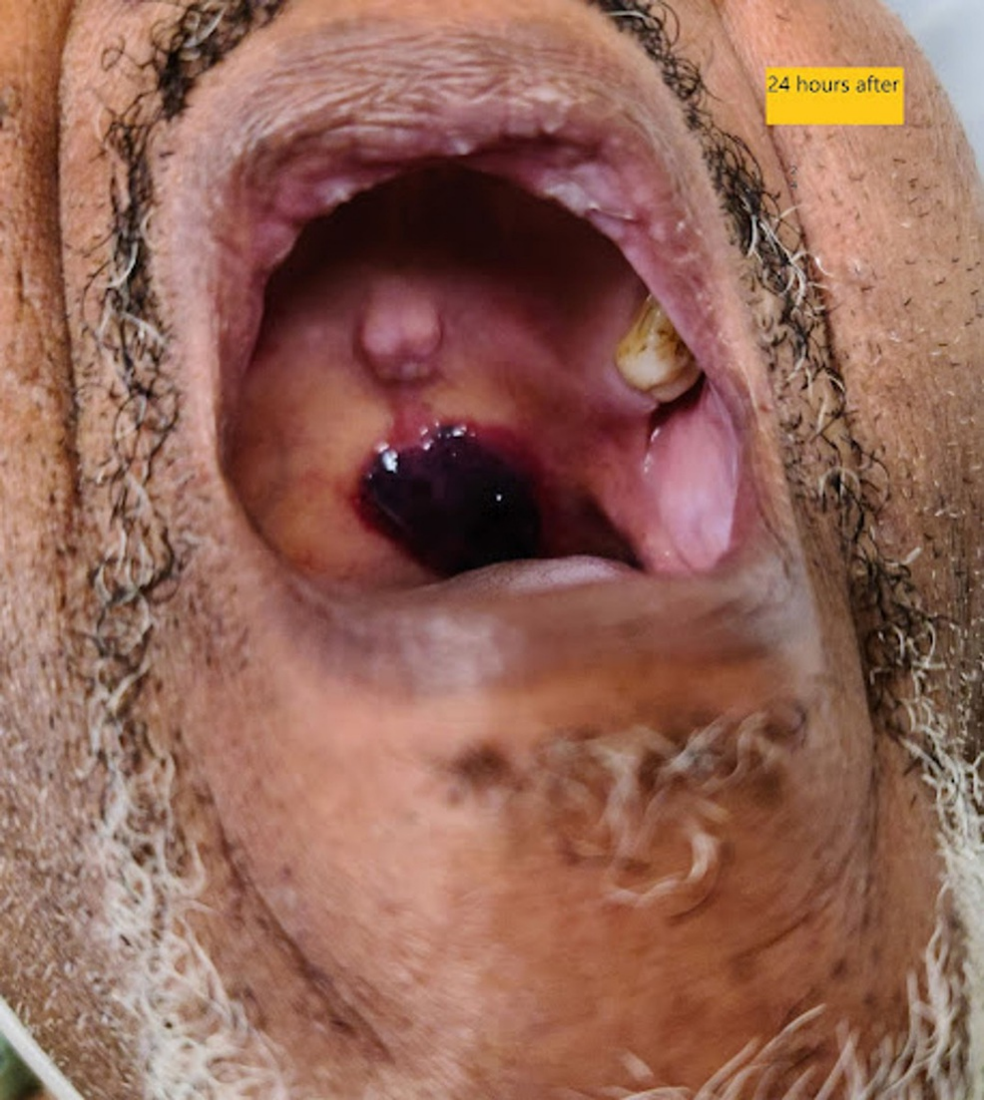
Marked Improvement Following Management With Solumedrol

## Discussion

The patient in Figure 1 had several risk factors for ABH: old age, hypertension, and recent oropharyngeal trauma (lunch) [6]. A diagnosis of ABH was established based on the proposed criteria by Ordioni et al., of which our patient met 6/9, including (I) and (II) [6] (Table 1).

**Table 1:** Diagnostic criteria for angina bullosa haemorrhagica (ABH) by Ordioni et al. (2019) [6]. For a positive diagnosis of ABH using these criteria, the case should meet a minimum of 6 out of 9 defined criteria, with criteria I and II as required.

Main criteria
(I) Clinically noticeable hemorrhagic bulla or erosion with a history of bleeding of the oral mucosa
(II) Exclusively oral or oropharyngeal localization
Additional criteria
(III) Palatal localization
(IV) Triggering event or promoting factor (food intake)
(V) Recurrent lesions
(VI) Favorable evolution without leaving a scar in a few days
(VII) Painless lesion, tingling, or burning sensation
(VIII) Normal platelet count and coagulation profile
(IX) Negative direct immunofluorescence

Our patient's presentation was typical, the blister enlarged posteriorly, causing a choking sensation that originally gave the condition "angina" as a name [7], this can lead to asphyxiation due to the size and localization (Figure 1). The most frequent locations for ABH include the soft palate, lateral border of the tongue, buccal mucosa, floor of the mouth, and oropharynx [6,8,9]. The proximity of the bullae to the larynx usually correlates with the risk for asphyxiation. Soft palatal and pharyngeal ABH are especially prone to causing airway obstruction, possibly due to the absence of hard supporting structures in these areas predisposing the bullae to unchecked expansion [10,11]. A case by Pahl et al. presented similarly but ultimately needed a surgical airway after failed attempts at intubation and worsening patient condition [10]. Some authors have even suggested pre-rupturing palatal and pharyngeal bullae to curb the risk of airway compromise [12].

Although inhaled steroids have been implicated as a possible risk factor for ABH, the role of steroids in the acute management of ABH has not been well represented in the literature. Steroids should be considered for use in the acute management of expanding blood-filled blisters with the potential of airway obstruction. The resolution of symptoms as seen in Figure 2 could be attributed to the anti-inflammatory effects of methylprednisolone and raises questions about the proposed pathogenesis of ABH. Inflammation is believed to play a minimal role in ABH, even though some studies have shown the background of chronic inflammatory cells in the lamina propria of biopsied lesions [8,9].

In our study, a biopsy of the blister and the perilesional area was not feasible due to the patient’s acute presentation and the risk for airway obstruction. Although not necessary for a diagnosis of ABH, a biopsy would have aided in ruling out other blistering conditions like pemphigus, mucosal pemphigoid, cicatricial pemphigoid, epidermolysis bullosa acquisita, linear IgA dermatosis, toxidermia, bullous lichen planus, erythema multiforme, oral amyloidosis, and fixed drug eruption that have similar presentations [12].

## Conclusions

There is poor literature on ABH due to its rareness and probably due to misdiagnosis. A high index of suspicion is required to make the diagnosis. While the condition is usually benign, adequate proactive measures should be made available in case airway obstruction is suspected. The specific mode of treatment is debatable. However, steroids might be helpful in the acute setting.
